# What is the status of immunotherapy in thyroid neoplasms?

**DOI:** 10.3389/fendo.2022.929091

**Published:** 2022-08-05

**Authors:** Alejandro Garcia-Alvarez, Jorge Hernando, Ana Carmona-Alonso, Jaume Capdevila

**Affiliations:** Vall d’Hebron University Hospital, Medical Oncology Department. Gastrointestinal and Endocrine Tumor Unit. Vall Hebron Institute of Oncology (VHIO), Barcelona, Spain

**Keywords:** thyroid neoplasms, immunotherapy, PD-L1, tumor mutational burden, tumor microenvironment

## Abstract

Immunotherapy has changed the treatment of patients with advanced cancer, with different phase III trials showing durable responses across different histologies. This review focuses on the preclinical and clinical evidence of potential predictive biomarkers of response and efficacy of immunotherapy in thyroid neoplasms. Programmed death-ligand 1 (PD-L1) staining by immunohistochemistry has shown higher expression in anaplastic thyroid cancer (ATC) compared to other subtypes. The tumor mutational burden in thyroid neoplasms is low but seems to be higher in ATC. Immune infiltrates in the tumor microenvironment (TME) differ between the different thyroid neoplasm subtypes. In general, differentiated thyroid cancer (DTC) has a higher number of tumor-associated lymphocytes and regulatory T cells (Tregs), while ATC and medullary thyroid cancer (MTC) display a high density of tumor-associated macrophages (TAMs). Nevertheless, results from clinical trials with immunotherapy as monotherapy or combinations have shown limited efficacy. Further investigation into new strategies aside from anti-cytotoxic T-lymphocyte antigen 4 (CTLA-4)/programmed death 1 (PD-1)/PD-L1 antibodies, validation of predictive biomarkers, and better population selection for clinical trials in thyroid neoplasms is more than needed in the near future.

## Introduction

Thyroid cancer is the most common endocrine malignancy. With 586,202 new cases and 43,646 deaths in 2020, it is the ninth most common malignancy diagnosed and the 24th most deadly malignancy (1). Its incidence has been growing by an average of 4.5% per year. Incidental detection of thyroid nodules on imaging studies may be one of the main reasons (2).

All thyroid cancers, except the medullary subtype, arise from follicular cells of the thyroid gland. Differentiated thyroid cancer (DTC) accounts for 88% of all thyroid neoplasms. DTC encompasses papillary thyroid carcinomas (PTCs), follicular thyroid carcinomas (FTCs), and Hürthle cell carcinomas (3). Most patients with DTC are diagnosed at an early stage and cured by the combination of surgery with or without radioactive iodine. DTC has a good prognosis with a 5-year survival of over 95% for PTC, the most common subtype of DTC (4).

Poorly differentiated thyroid carcinoma (PDTC) is a follicular cell-derived thyroid neoplasm that has both histomorphology features and clinical outcomes intermediate between DTC and anaplastic thyroid cancer (ATC) (3).

ATC represents 1%–2% of all thyroid malignancies (3). ATC is a rapidly progressive disease characterized by a high mitotic rate, lymphovascular invasion, and undifferentiated cell morphology (4). This aggressive behavior is translated into invasion of surrounding tissues and the worst prognosis among thyroid neoplasms, with a 5-year survival near 0% (4).

Systemic treatment options for patients with metastatic DTC refractory to radioactive iodine and ATC include tyrosine kinase inhibitors (sorafenib, lenvatinib, and cabozantinib) for DTC and combination of surgery (whenever possible, as complete as possible), radiotherapy, and chemotherapy for ATC (5). For BRAF-V600E mutant ATC, the combination of dabrafenib and trametinib has been proven to dramatically improve survival (6).

On the other hand, medullary thyroid cancer (MTC) is a rare malignancy originating from neural crest-derived parafollicular C cells. MTC accounts for 4% of all thyroid neoplasms but represents up to 13% of all thyroid cancer-related deaths. Surgical resection remains the only curative treatment for most MTC patients. However, almost 50% of patients will still have residual or recurrent disease even after aggressive treatment. In patients with locally advanced unresectable or metastatic MTC, vandetanib and cabozantinib represent the approved treatment options (7).

In those molecularly selected patients harboring RET fusions, RET mutations, or Neurotrophic tyrosine receptor kinase (NTRK) fusions, treatment with pralsetinib and selpercatinib (RET inhibitors, which are Food and Drug Administration (FDA)-approved options for advanced progressive MTC) or entrectinib and larotrectinib (NTRK inhibitors) must be considered (8).

Taking into account previous data, new therapeutic approaches are needed for patients with advanced thyroid cancer and for those with thyroid cancer refractory to standard treatments.

Immunotherapy is one of the most promising treatment strategies for patients with unresectable or metastatic cancers nowadays. Antibodies targeting cytotoxic T-lymphocyte antigen 4 (CTLA-4) and programmed death 1 (PD-1) and its ligand [programmed death-ligand 1 (PD-L1)] are the most consolidated options (9).

Different phase III trials have explored the safety and efficacy of anti-CTLA-4, anti-PD-1, and anti-PD-L1 antibodies as either monotherapy or combination with another immunotherapy antibody, targeted therapy, or chemotherapy. The results from these trials have led to the approval of immunotherapy agents across different histologies (10–13).

However, not all patients with the same histology or across histologies experience the same benefit (10–13). This is why we need to identify which patients will benefit more from immunotherapy antibodies. The most studied predictive biomarkers are PD-L1 expression in tumor cells and tumor mutational burden (TMB), but investigation is needed in this field in order to find better biomarkers.

As in many other histologies, clinical trials with immunotherapy antibodies have been conducted in thyroid neoplasms. This review focuses on the preclinical and clinical evidence of potential predictive biomarkers of response and efficacy of immunotherapy in thyroid neoplasms.

## Tumor programmed death-ligand 1 staining in thyroid cancer

PD-L1, or cluster of differentiation 274 (CD274), is a transmembrane protein involved in adaptive immune response suppression. PD-L1 could be found at the membrane of dendritic cells and myeloid-derived suppressor cells (MDSCs). Moreover, tumor cells can express PD-L1 at their membrane (14).

Under normal physiologic conditions, negative immune checkpoint regulators are needed to ensure proper immune response intensity, which is crucial to reducing the damage to surrounding normal tissues and avoiding autoimmune responses (15).

The expression of PD-L1 in tumor cells could occur through two mechanisms. The first mechanism is intrinsic immune resistance, where the upregulation of PD-L1 occurs through activation of an oncogene-associated pathway (14). Thyroid cancer cell lines with BRAF V600E mutation have higher PD-L1 mRNA expression compared to BRAF wild-type cell lines. Thus, BRAF activation may cause intrinsic immune resistance. Mitogen-activated protein kinase kinase (MEK) inhibition (independent of BRAF status) and BRAF inhibition (only in BRAF mutant) in cell lines showed a significant decrease in both phosphorilated form of the extracellular signal-regulated kinase (pERK) and PD-L1 protein expression (16). In fact, in a cohort of 33 PTCs published by Angell et al. (17), BRAF V600E mutation (present in 51.5% of samples) was significantly associated with higher PD-L1 and indolamine 2,3-dioxygenase (IDO) proteins. Another immune evasion mechanism is major histocompatibility complex (MHC) class I downregulation that was present in 76% of the PTC samples analyzed by Angell et al. (17), MHC-I downregulation was numerically higher in BRAF wild-type tumors but linked to Mitogen-Activated Protein Kinase (MAPK) signaling pathway activation and associated with lesser immune cell infiltration (18).

The second mechanism is known as adaptive immune resistance. Activation of T-cell immune response after interaction with tumor cells increases the production of interferon-gamma (IFN-γ), which stimulates PD-L1 membrane expression in tumor cells. Therefore, PD-L1 expression may represent an adaptive mechanism for evasion of T-cell cytotoxic activity (14).

This ligand has gained interest since the introduction of the anti-PD-1/anti-PD-L1 immunotherapy antibodies. Moreover, higher PD-L1 staining percentage by immunohistochemistry (IHC) in tumor cells has been associated with better efficacy of anti-PD-1/anti-PD-L1 antibodies in some tumors, such as non-small cell lung cancer (19).

In this scenario, PD-L1 expression at the membrane of tumor cells has been proposed as a possible biomarker for immunotherapy. PD-L1 staining has been investigated in thyroid neoplasms. The most relevant results published to date are summarized in this section.

Several working groups have published results about PD-L1 expression in DTC with positivity percentages ranging from 6.1% to 87.5%. Although analyzing a large number of samples, the use of different anti-PD-L1 antibody clones and positivity thresholds may be the cause for the previously reported wide positivity range. In this context, the true PD-L1 expression in DTC is still unclear (16, 20–22).

Three small cohorts of PDTCs have been reported with PD-L1 expression ≥1% in 0% (20) and 7.7% (23) with the SP142 and the 22C3 antibodies, respectively, and PD-L1 ≥1% in 25% with the E1L3N antibody (24). The first two studies also reported PD-L1 staining results in the surrounding immune cells with no expression of PD-L1 (20, 23).

Regarding ATC, six cohorts have been published to date reporting PD-L1 expression. Overall, PD-L1 positivity varies from 22% to 65%. Nonetheless, PD-L1 positivity seems to be higher in ATC than in DTCs or PDTCs (20, 23, 25–28).

Moreover, 5.1% of ATCs harbor PD-L1 gene amplification, which is between the 10 highest percentages across all tumors (29).

Finally, PD-L1 expression has also been explored in five series of MTCs with PD-L1 positivity in tumor cells ranging from 6.25% to 28.6% (27, 30–33). Three of the published series have used the SP263 antibody and the same positivity threshold (PD-L1 ≥1%) (30–32) with PD-L1 positivity from 6.25% (n = 16 by Bongiovanni et al. (31)) to 21.8% (n = 87 by Bi et al. (30)). The biggest series has been analyzed by Shi et al. (33) involving 201 thyroid surgical specimens, reporting a PD-L1 positivity of 14.4% (defined as a combined positivity score higher or equal to 1) with the 22C3 antibody.

The extent of PD-L1 staining was low (1%–5%) for all tumors in the series reported by Bongiovanni et al. (31) and Kemal et al. (32) A reanalysis of the series by Bongiovanni et al. (31), using the combined positive score (CPS) algorithm, yielded a PD-L1 positivity of 18.8% (similar percentage compared to the result obtained by Shi et al.).

TIM-3 expression was observed in 48% of patients. In the majority of cases (84.4%), T-cell immunoglobulin and mucin-domain containing-3 (TIM-3) expression was restricted to tumor cells. Other co-inhibitory receptors, such as Lymphocyte Activating 3 (LAG-3) and T Cell Immunoreceptor With Ig And ITIM Domains (TIGIT), were observed in a lesser percentage of cases (3% in both cases) (34).

**Table 1** summarizes the results of PD-L1 staining in thyroid malignancies previously discussed.

**Table 1:** PD-L1 positivity in tumor cells in different subtypes of thyroid cancer.

Differentiated Thyroid Cancer					
Study	Anti-PD-L1 antibody clone	Sample type	Number of samples	PD-L1 positivity threshold	PD-L1 positivity in tumor cells
Ahn et al., 2017 (20)	SP142	Surgical thyroid samples	PTC ➔ n=326 FTC ➔ n=66	≥1% and ≥5%	PTC ➔ 6.1% and 0.9% FTC ➔ 7.6% and 7.6%
Cunha et al., 2013 (21)	Ab82059	Thyroid biopsies or surgical samples	PTC ➔ n=254 FTC ➔ n=40	≥1%	PTC ➔ 82.5% FTC ➔ 87.5%
Chowdhury et al., 2016 (22)	E1L3N	Tumor biopsy	PTC ➔ n=185	NR	40%
Angell et al., 2014 (17)	4059	Thyroid samples	PTC ➔ n=33	NR	53%
**Poorly Differentiated Thyroid Cancer**					
**Study**	**Anti-PD-L1 antibody clone**	**Sample type**	**Number of samples**	**PD-L1 positivity threshold**	**PD-L1 positivity in tumor cells**
Ahn et al., 2017 (20)	SP142	Surgical thyroid samples	n=6	≥1% and ≥5%	0% and 0%
Cameselle-García et al., 2021 (23)	22C3	Surgical thyroid samples	n=11	≥1%	7.7%
Rosenbaum et al., 2018 (24)	E1L3N	Surgical thyroid samples	n=28	≥5%	25%
**Anaplastic Thyroid Cancer**					
**Study**	**Anti-PD-L1 antibody clone**	**Sample type**	**Number of samples**	**PD-L1 positivity threshold**	**PD-L1 positivity in tumor cells**
Ahn et al., 2017 (20)	SP142	Surgical thyroid samples	n=9	≥1% and ≥5%	22.2% and 22.2%
Cameselle-García et al., 2021 (23)	22C3	Surgical thyroid samples	n=15	≥1%	60%
Cantara et al., 2019 (25)	SP263	Surgical thyroid samples	n=20	≥25%	65%
Chintakuntlawar et al., 2017 (26)	E1L3N	Surgical thyroid samples	n=48	≥1%	27%
Zwaenepoel et al., 2017 (27)	E1L3N	Thyroid biopsies or surgical samples	n=49	≥5%	28.6%
Wu et al., 2015 (28)	B7-H1	Thyroid biopsies or surgical samples	n=13	NR	23%
**Medullary Thyroid Cancer**					
**Study**	**Anti-PD-L1 antibody clone**	**Sample type**	**Number of samples**	**PD-L1 positivity threshold**	**PD-L1 positivity in tumor cells**
Bi et al., 2019 (30)	SP263	Surgical thyroid samples	n=87	≥1%	21.8%
Bongiovanni et al., 2017 (31)	SP263	Surgical thyroid samples	n=16	≥1%	6.25%
Kemal et al., 2021 (32)	SP263	Not reported	n=41	≥1%	12.2%
Shi et al., 2019 (33)	22C3	Surgical thyroid samples	n=201	CPS ≥1%	14.4%
Zwaenepoel et al., 2017 (27)	E1L3N	Thyroid biopsies or surgical samples	n=49	≥5%	28.6%

PTC, Papillary thyroid cancer; FTC, Follicular thyroid cancer; CPS, Combined positive score.

## Tumor mutational burden in thyroid cancer

The TMB quantifies the number of somatic mutations in a tumor per megabase (Mb), thus simplifying the variety of mutational processes across tumor types into an average number of mutations (35).

A higher number of mutations increase the possibilities of modifying the structure of a native protein, thus creating a new antigen or neoantigen. These neoantigens may increase the visibility of the tumor toward the immune system. Nonetheless, the type of mutation (instead of the number of mutations) may determine the immunogenicity of the neoantigen. Frameshift mutations, insertions, and deletions generate more changes in the protein’s structure and increase immunogenicity compared to non-synonymous mutations (36).

In this scenario, it is not surprising that although TMB has been evaluated as a predictive biomarker of benefit from immunotherapy, conflicting results have been published to date.

Main data about TMB in thyroid cancer come from analysis of PTC samples. We have data from 402 tumor samples evaluated by whole-exome sequencing (WES) in the context of The Cancer Genome Atlas (TCGA) project. An average of 0.41 nonsynonymous mutations (mut)/Mb were seen, which is a low TMB compared to other histologies (such as melanoma or non-small cell lung cancer) (37). Analysis of data from 400 PTC tumor samples out of cBioPortal also showed a low TMB of 0.2 mut/Mb (range 0.03–2.05 mut/Mb). The predicted neoantigen burden was 1 (range 0–18), which is also low (38). Indeed, an analysis of 3,083 tumor–normal pairs across 27 tumor types by WES evidenced that thyroid cancer had the third lowest TMB (TMB between 0.1 and 1 mut/Mb), behind rhabdoid tumor and Ewing sarcoma (39).

The KEYNOTE-158 is a phase II multicohort trial that evaluated the safety and efficacy of pembrolizumab as monotherapy in different cohorts of uncommon neoplasms. Cohort I enrolled 223 patients with PTC and FTC. Median TMB of the cohort was 1.7 mut/Mb with six patients (2.7%) reaching the definition of TMB-high (>13 mut/Mb). In a subgroup of 80 patients with evaluable disease by RECIST 1.1 (efficacy population) in this cohort, Overall response rate (ORR) was 100% (2/2) for patients with TMB-high and 3.8% (3/78) for patients with non-TMB-high. These data point out that TMB could identify patients more likely to respond to immunotherapy in DTC (40).

In patients with a higher number of mutations, two main mechanisms have been found. The first mechanism is deficiency of DNA mismatch repair (MMR) and seems to be the most frequent mechanism. It is related to loss-of-function mutations in MMR genes (such as MLH1, MSH2, or MSH6) (41, 42). In the cohort of PDTCs and ATCs analyzed by Landa et al., 12% of ATCs and 2% of PDTCs harbored genomic alterations in DNA MMR genes. Those tumors had a significantly higher number of mutations (7.5 *vs*. 2 mut/Mb in PDTCs and 16.5 *vs*. 5 mut/Mb in ATCs) (41). In the cohort by Pozdeyev et al. (42), this mechanism was found to be independent of genetic alterations in BRAF, rapidly accelerated fibrosarcoma (RAS) isoforms, or RET. The second mechanism is associated with increased activity of Apolipoprotein B mRNA Editing Catalytic Polypeptide-like (APOBEC) deaminase enzymes. This mechanism has been seen only in tumor samples harboring BRAF V600E mutations (42).

Regarding ATCs, comparative data of ATC TMB compared to TMB in DTCs and PDTCs have been published. Pozdeyev et al. analyzed two cohorts of tumor samples (DTC n = 583 and ATC n = 196) by either MSK-IMPACT or FoundationOne next-generation sequencing (NGS) gene panels. ATCs harbored significantly more genetic alterations than DTCs (41). Landa et al. sequenced by NGS (MSK-IMPACT panel) 117 thyroid tumors, including 84 PDTCs and 33 ATCs. ATCs had significantly more mutations than PDTCs (median number of mutations 6 *vs*. 2 mut/Mb) (40). TMB analysis in a cohort of 113 tumor samples from DTC, PDTC, and ATC confirmed that TMB in ATC was higher than TMB in other subtypes. Moreover, somatic copy-number alterations were higher in ATC compared to DTC, highlighting that ATC may have greater genome instability than DTC and PDTC (43).

Finally, in MTC, there is evidence of a low number of mutations. Agrawal et al. (44) sequenced the coding exons of approximately 21,000 genes in 17 cases of sporadic MTC with a median of 17 ± 8.8 mutations per tumor (range 4–29).

TMB may not be the best predictive biomarker of response to immunotherapy antibodies. However, it should be taken into account together with PD-L1 expression as part of the multidimensional depiction of a patient’s neoplasia immune milieu.

## Immune cell infiltration in the tumor microenvironment of thyroid cancer

A tumor is a complex mass not only composed of tumor cells but also supported by fibroblasts, immune cells, vascularization, and other supporting tissues. These elements that intermingle with tumor cells are known as the tumor microenvironment (TME). This TME is dynamic, maintains close communication with tumor cells, and plays a key role in tumor progression and metastases (45).

Although immune cells at first are able to recognize cancer cells and eliminate them, tumor cells escape from the immune system, reducing their exposition to cytotoxic T cells (downregulating the expression of the MHC-I molecules and upregulating the expression of inhibitory ligands) and recruiting cells with immunosuppressive functions [such as MDSCs and regulatory T cells (Tregs)]. These changes of the immune milieu at the TME are known as cancer immunoediting (46).

Cytotoxic T cells express CD8 at their membrane and are able to kill tumor cells (if they are capable of recognizing them). Upon activation, they produce IFN-γ, which activates an antitumor immune response, but also upregulates the expression of PD-L1 in tumor cells (47).

Tregs exert immunosuppressive functions [*via* production of interleukin (IL)-10] and are enriched at the TME. The most important Tregs are recognized by the expression of FoxP3. Those cells are attracted to the TME due to the secretion of chemokines (mainly CCL17/22 and CXCL12) by tumor cells (48).

Other cells with immunosuppressive functions are MDSCs. MDSCs are immature myeloid cells that exhibit strong immunosuppressive activity. Circulating MDSC levels have been found to be increased in patients with ATC compared to controls. However, the lack of established immune markers to define this population in humans has limited the evaluation of this immune population in tumor samples (49).

Nonetheless, tumor-associated macrophages (TAMs) are the most abundant cell population at the TME. They can be subdivided into M1 and M2 TAMs. While M1 TAMs display antitumor functions, M2 TAMs promote immunosuppression and tumor progression through production of IL-10 and transforming growth factor-beta (TGF-β). Thyroid tumor cells are able to produce chemokines (p.e. CCL2) that attract monocyte-macrophages to the TME, where they will differentiate preferably to M2 subtype (50).

In this complex scenario, it is important to analyze the immune cell population at the TME in thyroid neoplasms in order to predict the most appealing immunotherapy strategy. Some of the most relevant studies involving immune cell characterization in the TME of thyroid neoplasms are depicted in this section.

In general, DTCs have a higher number of tumor-associated lymphocytes and Tregs and lesser number of TAMs compared to ATC and MTC. It is important to highlight that most of the studies analyzing the immune milieu of DTC have used PTC tumor samples (17, 21, 51–57).

Analysis from 100 PTC specimens revealed that 24% of them had tumor-associated lymphocytes in the absence of thyroiditis. To further define the lymphocyte subtypes, 10 PTC primary tumors were analyzed. CD4+ and CD8+ T cells represented 54%–83% and 13%–39%, respectively. Nonetheless, CD4+FoxP3+ Tregs constituted 12%–36% of CD4+ T cells and positively correlated with the presence of lymph node metastases (51).

Actually, in a series published by Gogali et al. (52), no differences in T-cell (either CD4+ or CD8+) infiltration were seen in PTC samples (n = 65) compared to goiters. However, a significantly greater Treg infiltration was observed that positively correlated with the stage of the disease (52).

French et al. analyzed by flow cytometry lymph nodes involved with PTC (n = 25) and compared them to uninvolved lymph nodes from the same patients. IFN-γ+ CD8+ T cells and CD4+ FoxP3+ Tregs were enriched in PTC lymph nodes. Despite the presence of Tregs, a high portion of CD4+ and CD8+ T cells were able to produce IFN-γ when activated *in vitro*. However, high levels of Treg cells were associated with signs of exhaustion in T cells (expression of PD-1 and CD27) (53).

This immune milieu might be tailored by oncogenic mutations. In the cohort by Angell et al. of 33 PTCs, those samples harboring BRAF V600E mutation (51.5% of samples) had significantly lower CD8+/FoxP3+ cell ratio. This fact highlights the intrinsic immune resistance mechanism driven by BRAF V600E mutation (17). However, in the cohort reported by Cunha et al. (21), higher mRNA levels of B7H1 (PD-L1 gene) were significantly associated with the presence of CD3+, CD4+, and FoxP3+ lymphocytes. Although the BRAF V600E mutation has been linked to higher PD-L1 expression in tumor cells, the results from the cohort from Angell et al. (17) may represent an example of intrinsic immune resistance; whereas the results from the cohort from Cunha et al. (21), an example of adaptive immune resistance.

On the other hand, in DTC and PDTC, TAMs are found at lower proportions located within the lumen of follicles and mixed with tumor cells. In a series from the Memorial Sloan Kettering Cancer Center (MSKCC) tumor biopsies from 33 DTCs, 37 PDTCs and 20 ATCs were stained for CD68. High TAM density (≥10 CD68+ TAMs/0.28 mm^2^) was found in 27%, 54%, and 95% of DTCs, PDTCs, and ATCs, respectively (54).

In a different cohort of 36 PTCs with lymph node metastases, CD68+ cells accounted for 5%–75% of all cells. Higher TAM densities (≥25 CD68+ TAMs/section) were associated with larger tumors (55). Qing et al. analyzed 103 samples from PTCs and found an average density of CD68+ and CD163+ TAMs of 26 ± 19 TAMs/field, significantly higher than samples from goiters or follicular adenomas. IL-10 expression level was also determined (by RT-PCR), finding significantly higher levels compared to peripheral blood monocytes (56).

This immune population may have relation to the genotype of the tumor cells. Na et al. studied the immune cell population and the differentiation of 505 samples of PTC using data from TCGA project. Expression of differentiation thyroid genes was negatively correlated with myeloid cell infiltration, Treg infiltration, and expression of inhibitory immune checkpoint ligands (CTLA-4 and PD-L1). *BRAF-V600E* mutation (identified in 46.5% of patients) was associated with low expression of differentiation thyroid genes. Other series with a lower number of samples analyzed have obtained different associations. However, *BRAF-V600E* mutation may lead to the upregulation of key genes involved in the innate immune response and may be associated with higher levels of immune-suppressive molecules (57).

ATCs display a high density of TAMs and with lower counts of lymphocytes compared to DTCs. In a cohort of 27 ATC biopsies, TAMs (CD68+, CD163+, and NOX2+) were present in all samples with a mean proportion of 57% of the total cells. However, TAMs were distributed, forming networks, enfolding isolated tumor cells, and even separating them from blood vessels. Moreover, a high expression of connexins (Cx43) was found between TAMs. These findings raise the hypothesis that TAMs coordinate their intracellular metabolism and directly communicate blood vessels with tumor cells through this TAM network (58).

Analysis of the CD3+ lymphocytes in the TME showed a 55% positivity out of 27 samples in the previously presented cohort by Zwaenepoel et al. (27) In the analysis by Cameselle-García et al. (23), the majority of CD3+ cells were CD8+ (cytotoxic) T lymphoid population and S100+ dendritic cells with a minority of helper (CD4+) T lymphocytes at the interface of tumor/normal thyroid tissue. In fact, in the cohort by Zwaenepoel et al. (27), 30.6% of samples showed an excluded immune infiltrate and 14.3% displayed an immune-desert phenotype.

Finally, MTC TME is mainly characterized by few tumor-infiltrating immune cells. Multispectral imaging from 46 MTC archived surgical samples showed that CD68+/CD163+ TAMs were the most predominant myeloid cells at the TME. They were largely negative for MHC-II expression, which is a sign of immaturity and suggestive of M2 phenotype. CD8+ T cells were the predominant T-cell subtype at the TME and reached up to 18% and 38% of all non-tumor cells in primary tumor and lymph node metastases, respectively (59).

Moreover, PD-1+CD8+ T cells were present at higher densities in 22% and 32% of primary tumor and lymph node metastases, respectively (59). These data are similar to a PD-1 positivity in 25.3% of tumor-infiltrating immune cells reported by Bongiovanni et al. (31) Although PD-1 is expressed in immune cells, IFN-γ signatures (by RNA sequencing) were enriched in the majority of MTC samples and MHC-I expression was preserved in tumor cells in 89% of primary tumors and 62% of lymph node metastases (59).

As it has been described in this section, the TME is different for each thyroid neoplasm and its composition and relation to other immune cells, non-immune cells and tumor cells will determine its cytotoxic or immunosuppressive activity.

## Immunotherapy in thyroid cancer: results from clinical trials

Immunotherapy, either as monotherapy, dual therapy, or combination with other agents, has been explored in different clinical trials in patients with thyroid neoplasms. To date, results from clinical trials assessing the efficacy of pembrolizumab and spartalizumab in monotherapy have been communicated.

The phase Ib KEYNOTE-028 trial assessed the efficacy of pembrolizumab in patients with PD-L1+ (membranous staining on ≥1% with the 22C3 antibody) locally advanced or metastatic FTC or PTC. Out of 51 patients screened, 71% (n = 36) met the positivity for PD-L1 staining. Finally, 22 patients were enrolled. Pembrolizumab achieved an ORR of 9% and median Progression-free survival (PFS) of 7 months (60).

The efficacy of spartalizumab was investigated in a phase I/II clinical trial for patients with ATC. The results of the phase II part of the study have been published. Forty-two patients were enrolled, achieving an ORR of 19% (including 7% CR) by RECIST 1.1 (ORR increased to 24% by irRC response criteria) and a PFS of 1.7 months. All patients with CR had BRAF wild-type tumors (61).

Biomarker analysis was performed on 40 tumor samples. PD-L1 positivity threshold (≥1% positive in tumor cells with the 22C3 antibody) was met for 70% of patients. Moreover, PD-L1 positivity was associated with better ORR, PFS, and OS, with statistically significant differences reached only for ORR. ORR was also numerically higher in patients with ≥1% CD8 baseline expression (61).

Finally, baseline IFN-γ signature by RNA sequencing was obtained from 18 patients. Correlation was found between the best percentage change of target lesions by RECIST criteria and IFN-γ signature (61).

To date, only one trial has investigated the efficacy of immunotherapy in combination. A phase II trial investigated the combination of nivolumab and ipilimumab in three different cohorts: RAIR DTC (n = 32, including 4 patients with PDTC) and two additional exploratory cohorts (10 patients with ATC and 7 patients with MTC). ORR for each of the three cohorts was 9.4%. Results from other clinical trials assessing the efficacy of immune checkpoint inhibitors in combination are awaited (62).

Although immunotherapy, either as monotherapy or as combination, was well tolerated with any safety concerns compared to previous trials in different tumor origins, it seems to offer limited antitumor efficacy in patients with advanced and refractory thyroid neoplasm. In this setting, combination of immunotherapy with antiangiogenic agents may represent an interesting option.

Neoangiogenesis is increased in thyroid neoplasms and partially promoted by an increase in the levels of vascular endothelial growth factor (VEGF). *VEGF-A* mRNA overexpression has been found in 51% of PTC (n = 62), 80% of PTC lymph node metastases (n = 32), and 85% of undifferentiated thyroid carcinomas (n = 11). This mRNA overexpression leads to VEGF-A protein overexpression, which is significantly higher in undifferentiated thyroid carcinomas compared to PTC. VEGF is produced not only by tumor cells but also by immune cells that infiltrate the TME (63).

VEGF-A is a protein capable of inducing neoangiogenesis at TME but also has immunosuppressive properties. As an example, CD8+ T cells express VEGF-R1 and VEGF-R2 receptors and Tregs express VEGF-R2 receptors. Those receptors, upon activation in immune cells, can lead to T-cell exhaustion and Treg induction (64).

Taking into account the previous data, modulation of VEGF-A may have an impact on immune cell population at the TME. A mouse model of colorectal cancer (CT26) treated with anti-VEGF-A agents (either antibodies or tyrosine kinase inhibitors) resulted in the reduction of PD-1 expression on intratumoral CD8+ T cells, restoration of IFN-γ production by those CD8+ T cells, and reduction in the number of Tregs in the spleen (65).

These observations have been partially seen in humans. In patients with colorectal cancer treated with the combination of bevacizumab and chemotherapy, peripheral blood Treg percentage was significantly reduced after two cycles (65).

No data to date have been published in patients with thyroid cancer. Nonetheless, two different trials have been reported to date investigating the combination of immune checkpoint blockade with lenvatinib, a multikinase inhibitor with antiangiogenic activity.

Retrospective data from a German center involving a cohort of eight patients with metastatic ATC (n = 6) or PDTC (n = 2) who had progressed to chemotherapy treated with lenvatinib and pembrolizumab have been published. All patients were BRAF V600E wild type. A very promising ORR of 75% and a median PFS of 17.4 months were observed (66).

These results justified the development of the ATLEP phase II trial evaluating this combination in patients with BRAF V600E wild-type PDTC and ATC. Interim results from a cohort of 36 evaluable patients (29 patients with ATC and 7 patients with PDTC) were communicated at the 2021 Annual Meeting of the American Thyroid Association annual meeting. ORR at 3 months (primary endpoint) was 38.5% among the first 26 patients. Only one patient with ATC had progressive disease as best response at 3 months (67).

Additionally, in the single-center retrospective cohort previously stated, a biomarker analysis was also performed. All tumors were PD-L1+ (using the SP263 clone antibody) with a tumor proportion score (TPS; proportion of PD-L1+ tumor cells of 100 tumor cells) ranging from 1% to 90% and a CPS (number of PD-L1+ tumor and immune cells within 100 tumor cells) ranging from 5 to 100. TMB (assessed by WES) ranged in 7/8 patients from 3 to 81.87 mut/Mb, with two patients with a TMB ≥10 mut/Mb. Interestingly, long-term remissions over 2 years all had a TPS above 50%, a CPS higher than 75, and/or a TMB >5/MB (66).

The combination of lenvatinib with pembrolizumab has also been explored in a single-arm phase II trial in patients with radioiodine-refractory DTC. Preliminary results were communicated at the 2020 ASCO annual meeting. Of 29 evaluable patients, the combination achieved an ORR of 62% (with no CR) and a PFS at 12 months of 74% (68).

Among ongoing clinical trials for thyroid neoplasms, some are testing the combination of MAPK inhibitors with anti-PD-1/PD-L1 inhibitors. There is *in vitro* and *in vivo* evidence supporting the development for this combination.

In an immunocompetent murine model of orthotopic ATC, treatment with BRAF inhibitor PLX4720 alone, in combination with anti-PD-1, or with anti-PD-L1 antibody, led to significant tumor reduction of 41%, 61%, and 64%, respectively. In fact, overall survival of the combination arms was significantly longer compared to PLX4720 alone. While treatment with PLX4720 or anti-PD-1/PD-L1 antibody increased the CD8+ T-cell infiltration and treatment with PLX4720 increased Natural killer (NK) cell and decreased MDSC infiltration, the combination of both treatments improved the previous findings and decreased Ki67 staining, increased granzyme B staining, and increased IFN-γ production (69).

**Figure 1:**
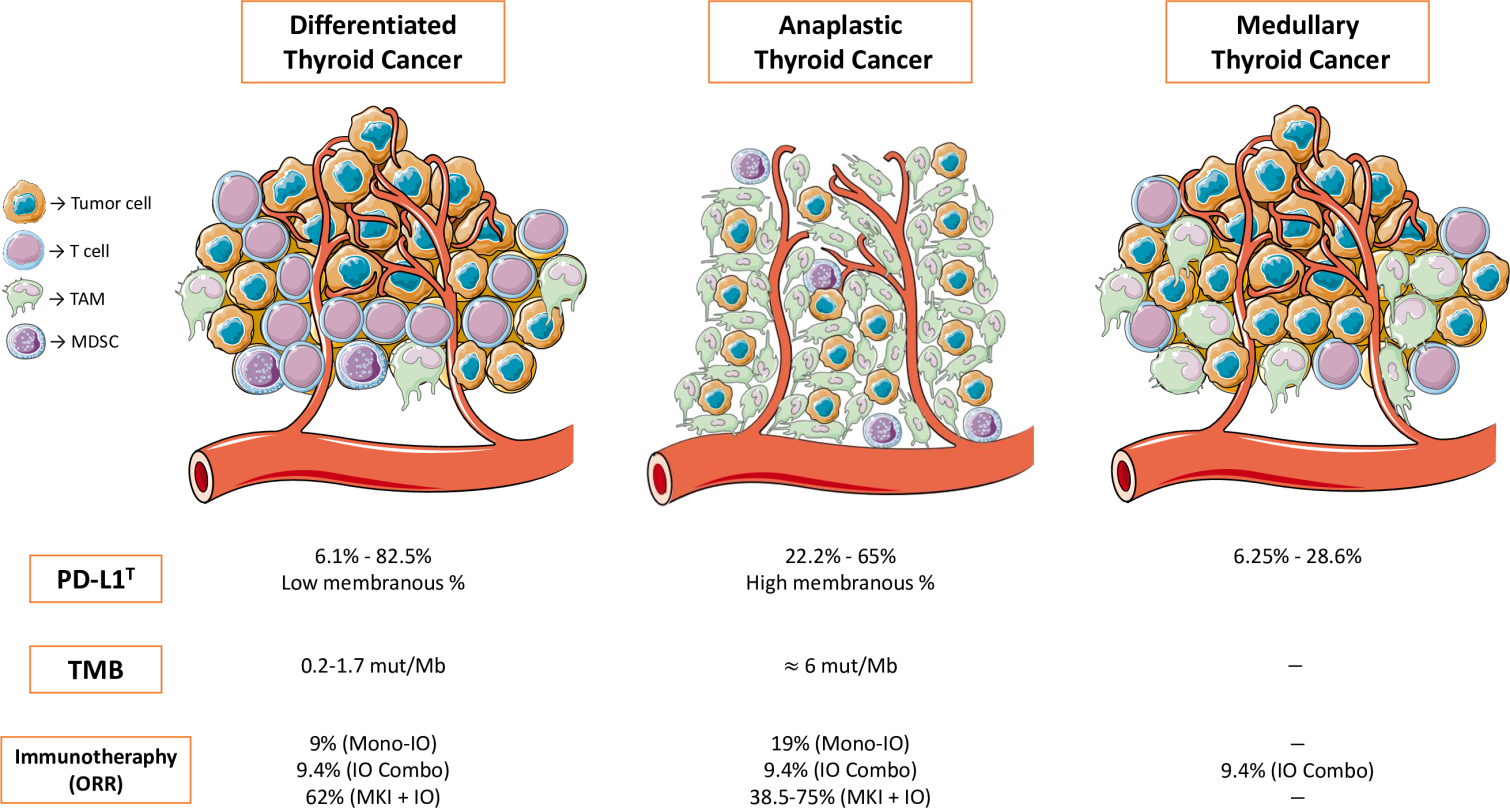
Summary of the most relevant information about biomarkers and immunotherapy efficacy in clinical trials in thyroid neoplasms. TAM, tumor-associated macrophage; MDSC, myeloid-derived suppressor cell; PD-L1^T^, PD-L1 membranous expression in tumor cells; TMB, tumor mutational burden; ORR, overall response rate; Mono-IO, immunotherapy monotherapy; IO-Combo, immunotherapy in combination with immunotherapy; MKI + IO, multikinase inhibitor in combination with immunotherapy.

Some of the most interesting clinical trials in progress involving immunotherapy are summarized in **Table 2** (70–72). **Figure 1** summarizes the most relevant information about biomarkers and immunotherapy efficacy in clinical trials in thyroid neoplasms previously developed.

**Table 2:** Ongoing clinical trials involving immunotherapy and combinations with immunotherapy in thyroid neoplasms.

Clinicaltrials.gov Identifier	Title	Phase	N	Population	Treatment Arms	Primary Endpoint
NCT03753919 (70)	A Phase II Study of Durvalumab (MEDI4736) Plus Tremelimumab for the Treatment of Patients With Progressive, Refractory Advanced Thyroid Carcinoma - The DUTHY Trial.	II	46	**- Cohort 1:** Advanced, radioiodine-refractory differentiated thyroid carcinoma, including papillary, follicular, Hürthle. Cell and poorly differentiated thyroid carcinoma (DTC). **- Cohort 2:** Advanced medullary thyroid carcinoma (MTC). **- Cohort 3:** Advanced anaplastic thyroid cancer (ATC).	Durvalumab (1,500 mg) plus tremelimumab (75 mg) every 4 weeks up to 4 cycles followed by durvalumab (1500 mg) every 4 weeks.	PFS at 6 months and OS at 6 months.
NCT04400474 (71)	Exploratory Basket Trial of Cabozantinib Plus Atezolizumab in Advanced and Progressive Neoplasms of the Endocrine System. CABATEN Study.	II	144 (all cohorts)	**Cohort 2:** Anaplastic thyroid cancer in first-line or after progression to chemotherapy or investigational drugs.	Cabozantinib (40 mg tablets, oral, once daily) + Atezolizumab (1,200 mg intravenously, every 3 weeks).	ORR
NCT04560127	A Single-arm, Non-randomized, Single-center Study to Evaluate Camrelizumab in Combination With Apatinib in Patients With Radioactive Iodine-refractory Differentiated Thyroid Cancer.	II	10	Locally advanced or metastatic differentiated thyroid cancer (papillary, follicular, Hürthle cells, poorly differentiated carcinoma).	Apatinib (250 mg PO QD) + Camrelizumab (200 mg, i.v., every 2 weeks).	PFS
NCT03914300	Phase II Study of XL184 (Cabozantinib) in Combination With Nivolumab and Ipilimumab (CaboNivoIpi) in Patients With Radioiodine-Refractory Differentiated Thyroid Cancer Whose Cancer Progressed After One Prior VEGFR-Targeted Therapy.	II	24	Radioactive iodine (RAI)-refractory/resistant papillary thyroid cancer (PTC), follicular thyroid cancer (FTC), or Hürthle cell thyroid cancer (HTC). The patient’s disease must have progressed on one line of VEGFR-targeted therapy (including, but not limited to, sorafenib, sunitinib, vandetanib, pazopanib, or lenvatinib)	Cabozantinib + Nivolumab + Ipilimumab.	ORR
NCT03215095	Radioiodine (RAI) in Combination With Durvalumab (Medi4736) for RAI-avid, Recurrent/Metastatic Thyroid Cancers.	I	11	Thyroid carcinoma of follicular origin (papillary, follicular, Hürthle cell or poorly differentiated) and at least one RAI-avid lesion identified on the radioiodine scan.	Durvalumab (1,500 mg i.v. every 4 weeks) + Radioiodine (100 mCi).	DLT
NCT03122496	A Pilot Study of Durvalumab (MEDI4736) With Tremelimumab in Combination With Image Guided Stereotactic Body Radiotherapy (SBRT) in the Treatment of Metastatic Anaplastic Thyroid Cancer.	I	13	Anaplastic thyroid cancer with clinical evidence of metastatic disease not curable by either surgery or radiation therapy	Durvalumab + Tremelimumab combined with SBRT (9Gy x 3 given within 2 weeks after the completion of cycle 1 of durvalumab and tremelimumab).	OS at 1 year
NCT03360890	Synergy of Pembrolizumab Anti-PD-1 Immunotherapy With Chemotherapy for Poorly Chemo-responsive Thyroid and Salivary Gland Tumors. The iPRIME Study.	II	46	Cohort B: Thyroid cancer, RAI-refractory and after failure, intolerance to or refusal of anti-antiangiogenic therapy, or with evidence of dedifferentiated or anaplastic histology.	Pembrolizumab (*via* i.v. at 200 mg every 3 weeks) + Docetaxel (*via* i.v. at 75 mg/m^2^ every 3 weeks for 3–6 cycles).	RR
NCT03246958	A Phase 2 Study of Nivolumab Plus Ipilimumab in RAI Refractory, Aggressive Thyroid Cancer With Exploratory Cohorts in Medullary and Anaplastic Thyroid Cancer.	II	53	Metastatic, RAI refractory, differentiated thyroid cancer (including papillary and follicular thyroid cancer and poorly differentiated thyroid cancer), with progression within 13 months prior to study registration.	Ipilimumb + Nivolumab.	RR
NCT02973997	Combination Targeted Therapy With Pembrolizumab and Lenvatinib in Progressive, Radioiodine-Refractory Differentiated Thyroid Cancers: A Phase II Study.	II	60	Locally recurrent and unresectable and/or distant metastatic DTC (including papillary and follicular thyroid cancer and poorly differentiated thyroid cancer)	Pembrolizumab + Lenvatinib.	Complete response rate
NCT04061980	Encorafenib/Binimetinib With or Without Nivolumab for Patients With Metastatic BRAF V600 Mutant Thyroid Cancer.	II	40	Histologically (or cytologically) confirmed diagnosis of metastatic, radioiodine (RAI) refractory, BRAFV600E/M mutant differentiated thyroid cancer (DTC)	Arm I: Encorafenib + Binimetinib Arm II: Encorafenib + Binimetinib + Nivolumab.	ORR
NCT04171622	Lenvatinib in Combination With Pembrolizumab for Stage IVB Locally Advanced and Unresectable or Stage IVC Metastatic Anaplastic Thyroid Cancer.	II	25	Unresectable or metastatic anaplastic thyroid carcinoma. Patients with a BRAFV600E mutation, who are unable to dabrafenib/trametinib, are eligible.	Pembrolizumab + Lenvatinib.	OS, PFS, and RR
NCT03181100	Atezolizumab Combinations With Chemotherapy for Anaplastic and Poorly Differentiated Thyroid Carcinomas.	II	50	Unresectable or metastatic anaplastic thyroid or poorly differentiated thyroid carcinomas.	**Cohort I:** Vemurafenib + Cobimetinib + Atezolizumab. **Cohort II:** Cobimetinib + Atezolizumab. **Cohort III:** Atezolizumab + Bevacizumab. **Cohort IV:** Paclitaxel or Nab-Paclitaxel + Atezolizumab.	OS
NCT04675710	Pembrolizumab in Combination With Dabrafenib and Trametinib as a Neoadjuvant Strategy Prior to Surgery in BRAF-Mutated Anaplastic Thyroid Cancer.	II	30	BRAFV600E mutation-positive anaplastic thyroid carcinoma surgically resectable.	Dabrafenib + Trametinib + Pembrolizumab.	Complete gross surgical resection (R0 or R1 resection) and OS

N, number of patients expected to be enrolled; PFS, progression-free survival; ORR, overall response rate; DLT, dose-limiting toxicity; OS, overall survival; RR, response rate.

## Conclusions

Results from clinical trials with the use of immunotherapy have shown modest activity in DTC and MTC. However, ATC seems to benefit from immune checkpoint inhibitors with an ORR ranging from 19% to 75%, with some patients achieving CR. Further investigation in those thyroid neoplasm subtypes with better results is desirable in order to broaden the percentage of patients benefiting from immunotherapy or combinations.

Although PD-L1 expression, TMB, and immune infiltrate have been discussed as possible biomarkers, their predictive role in thyroid neoplasms is still unknown. The role of other biomarkers, such as gene signatures, predicting the probability of immune cellular stimulation, or the immunosuppressive activity at the TME will be necessary across all tumors in order to select those patients with a higher probability of responding to immunotherapies.

Deeper knowledge of the immune milieu of thyroid cancer, strong predictive biomarkers (or combination of existing ones), and better clinical trial strategies for each thyroid neoplasm subtype are more than needed in the near future.

## Author contributions

AG-A selected the articles to review, designed and wrote the article. JH, AC-A and JC reviewed the article. All authors contributed to the article and approved the submitted version.

## Conflict of interest

AG-A reports Speaker’ Bureau from Angellini and Travel-Accommodations, Expenses from Eisai, Ipsen and Pfizer. JH reports scientific consultancy role for Eisai, Ipsen and Pfizer. EG reports a scientific consultancy role for Merck Serono, Pfizer, Novartis, Bayer, Eisai, Ipsen, Adacap and Sanofi. - JC reports: Personal conflicts of interest: Scientific consultancy role (speaker and advisory roles) from Novartis, Pfizer, Ipsen, Exelixis, Bayer, Eisai, Advanced Accelerator Applications, Amgen, Sanofi, Lilly, Hudchinson Pharma, ITM, Advanz, Merck Serono, Esteve. Research support: Research grants from Novartis, Pfizer, Astrazeneca, Advanced Accelerator Applications, Eisai, Amgen and Bayer. ARG reports scientific consultancy role for Novartis, Sanofi, Bayer, Amgen, Valentech, Baxalta and Novo Nordisk.

The remaining author declare that the research was conducted in the absence of any commercial or financial relationships that could be construed as a potential conflict of interest.

## Publisher’s note

All claims expressed in this article are solely those of the authors and do not necessarily represent those of their affiliated organizations, or those of the publisher, the editors and the reviewers. Any product that may be evaluated in this article, or claim that may be made by its manufacturer, is not guaranteed or endorsed by the publisher.
